# Body Image Problems and Disordered Eating Behaviors in Italian Adolescents With and Without Type 1 Diabetes: An Examination With a Gender-Specific Body Image Measure

**DOI:** 10.3389/fpsyg.2020.556520

**Published:** 2020-09-23

**Authors:** Alda Troncone, Crescenzo Cascella, Antonietta Chianese, Angela Zanfardino, Alessia Piscopo, Anna Borriello, Francesca Casaburo, Emanuele Miraglia del Giudice, Dario Iafusco

**Affiliations:** ^1^Department of Psychology, University of Campania “Luigi Vanvitelli”, Caserta, Italy; ^2^Department of Woman, Child and General and Specialized Surgery, University of Campania “Luigi Vanvitelli”, Napoli, Italy

**Keywords:** type 1 diabetes, adolescence, disordered eating behaviors, body image, gender, weight

## Abstract

**Objective:**

To examine body image problems and their associations with disordered eating behavior in adolescents with type 1 diabetes and well-matched healthy peers.

**Methods:**

Using a cross-sectional design, 183 adolescents with type 1 diabetes (13.02–18.05 years) were recruited from diabetes centers in southern Italy and compared to healthy peers matched for age and gender. Participants completed self-report measures of disordered eating behaviors (DEPS-r and EDI-3RF) and a gender-specific body image problem questionnaire (SATAQ-4R). Socio-demographic and clinical data (zBMI, HbA1c, and disease duration) were also collected. Hierarchical multiple linear regression analyses were computed to determine the relative importance of diabetes variables and body image problems on participants’ disordered eating behaviors after controlling for demographic variables.

**Results:**

Adolescents with type 1 diabetes showed diabetes-specific eating problems in 37.7% of cases and had more eating problem symptoms (assessed as drive for thinness and bulimia) than healthy peers. Male adolescents with type 1 diabetes did not display more body image problems (*p* > 0.05); females with type 1 diabetes compared to females in the control group were found to be more pressured by family (*p* = 0.025) but less by media (*p* = 0.022) to improve their appearance and attain a thin body. zBMI and body image problems contributed to a significant increase in disordered eating behavior risk both in male and female adolescents with diabetes and in healthy peers (zBMI 0.213 < β < 0.426, *p* < 0.05; body image 0.243 < β < 0.572, *p* < 0.05). None of the variables analyzed were found to significantly predict male bulimic symptoms (all β < 0.296, *p* > 0.05).

**Conclusion:**

Since in adolescence type 1 diabetes and insulin therapy may increase the risk of weight gain and promote focus and attention on the body and thus contribute to the development of body image problems and disordered eating behaviors, continuity of medical, nutritional, and psychological care is needed.

## Introduction

Type 1 diabetes mellitus (T1D) is one of the most common chronic diseases of childhood (Stanescu et al., 2012; Mayer-Davis et al., 2017), one in which an autoimmune destruction of pancreatic β-cells leads to an absolute insulin deficiency (Todd, 2010; Atkinson et al., 2014). The T1D treatment regimen is complex and multifaceted, and it entails, from the time of diagnosis, the adherence of the patient (and his/her family) to many disease specific behaviors, such as lifelong insulin replacement therapy, blood glucose monitoring, dietary management, physical activity, and exercise (Fairchild, 2015; National Institute for Health and Care Excellence, 2015; American Diabetes Association [ADA], 2020b).

Type 1 diabetes and its management requires considerable changes in the lifestyle of the child and often has a serious effect on the patient’s psychosocial well-being, so much so that several studies have shown that children with T1D are at higher risk to develop mental health problems (Cameron and Northam, 2012; Hagger et al., 2016; Delamater et al., 2018), which in turn may negatively influence the course and prognosis of T1D (Bernstein et al., 2013).

In particular, according to most recent literature reviews (Hanlan et al., 2013; Conviser et al., 2018; Pursey et al., 2020), disordered eating behaviors (DEBs) were more common in youths with T1D compared to healthy peers, and this was significantly associated with poorer glycemic control (Young et al., 2013).

Disordered eating behaviors often manifest in a multitude of ways, including mild/more extreme dieting behavior with caloric restriction, unhealthy dieting through skipping meals, binge eating attacks, and compensatory behaviors for weight control such as self-induced vomiting, excessive exercise, and use of insulin restriction for intentional caloric purging (Neumark-Sztainer, 1996; Olmsted et al., 2008).

Among possible predictors, body image problems have been consistently recognized as a key contributor to DEB onset both in the general population (Striegel-Moore and Debra, 2002; Fairburn and Harrison, 2003; Striegel-Moore and Bulik, 2007; Amaral and Ferreira, 2017; Girard et al., 2018) and in adolescents with chronic illness (Neumark-Sztainer et al., 2002).

Similarly, empirical evidence has shown that in youths and adults with T1D, the level of satisfaction with their perceptions of body image (along with other factors such as BMI, global and physical appearance-based self-worth, and depression), weight, and shape were associated with higher levels of DEB (Kichler et al., 2008; Troncone et al., 2016, 2018; Verbist and Condon, 2019). Body image dissatisfaction was described to be a significant predictor of eating problems in adolescents and young adult participants with T1D (Falcão and Francisco, 2017; Araia et al., 2020). In addition, adolescent girls with T1D and eating disorders had more negative body self-images than girls with T1D but without eating problems (Grylli et al., 2010), and youths with T1D who manipulated insulin as a means of weight control were described as more likely to report body dissatisfaction than youths with T1D who did not manipulate insulin (Ackard et al., 2008; Olmsted et al., 2008).

Theoretical models of DEB risk in T1D, such as the Modified Dual Pathway (Peterson et al., 2018; Rancourt et al., 2019), also recognized the role of body dissatisfaction along with the physiological disease processes and treatment-based factors (i.e., weight gain resulting from insulin therapy, hunger/satiety dysregulation, frequent blood glucose fluctuations, dietary regimen), or some combination of them, in predisposing youth with T1D to engage in unhealthy compensatory weight control behaviors (e.g., bulimic symptoms).

Studies analyzing gender differences suggested that adolescent females with T1D showed more DEBs than male adolescents (males 8.6–11.7% vs. females 27.7–59.2%) (Wisting et al., 2013; Baechle et al., 2014; Colton et al., 2015), as also described in the general population (males 2.2–34.8% vs. females 8.4–54.6%) (Neumark-Sztainer et al., 2011). Females with T1D also showed greater body image dissatisfaction than males (Araia et al., 2017). Societal pressure to conform to a “thin ideal” and pubertal/developmental factors (e.g., peripubertal weight gain causing higher body dissatisfaction) were proposed as explanations for body dissatisfaction and related higher prevalence of DEB in girls and young women compared to young men with T1D (Young-Hyman and Davis, 2010; Broadley et al., 2020).

In all, it should be noted that the literature examining the association between body image problems and DEB in the T1D population still remains somewhat conflicting and seems to present several limitations.

First, there is no general agreement that body image problems are always reported in youths with T1D. Specific elements of the illness and its treatment, -i.e., dietary restrictions, recurring weight variation, the subjective perception of living in an unhealthy body, focus and attention on the body, daily need for injections, etc.- are thought to facilitate the development of a negative body image (Colton et al., 1999; Shaban, 2010). Greater body dissatisfaction and an attitude toward body image problems have been reported in several studies on adolescents and young adults with T1D (Neumark-Sztainer et al., 2002; Kichler et al., 2008; Kaminsky and Dewey, 2013, 2014; Pinquart, 2013; Troncone et al., 2016, 2018). However, other evidence showed that adolescents with T1D had healthy body images and found no significant differences between participants (young adults and adolescents) with and without T1D in body image dissatisfaction or in their self-reports of body image (Meltzer et al., 2001; Ackard et al., 2008; Kaminsky and Dewey, 2013; Falcão and Francisco, 2017).

Second, as highlighted by Smith et al. (2020), little work in T1D research has examined body image problems and their associations with DEB while taking into account gender differences. In the majority of T1D studies, the body image problems have been measured as weight or body dissatisfaction and consequently evaluated by using self-report measures. Specifically, some measures consisted of items assessing dissatisfaction with body shape, weight and shape concerns, level of satisfaction with their physical appearance and significance of physical appearance (Kichler et al., 2008; Olmsted et al., 2008; Gawlik et al., 2016; Verbist and Condon, 2019). Other measures involved gender-specific silhouettes and merely evaluated the participants’ levels of satisfaction with their weight and physical appearance by measuring differences between perceived actual size and ideal size (Troncone et al., 2016, 2018; Araia et al., 2017, 2020; Falcão and Francisco, 2017). It should be pointed out, as emphasized by Pursey et al.’s review (2020) about eating disorder measures, that a gender bias across body image measures can be identified: tools are often focused on body image concerns more frequently reported among females compared to males (e.g., drive for thinness, belief of being fat, weight loss concerns), with items specifically evaluating concerns salient for females (e.g., concerns about specific aspect/shape of body parts such as thighs, hips, etc.) and less (if at all) focused on symptoms recognized as more central to males, such as desire for a muscular and athletic physique, concern about muscle mass, salience of muscular appearance, muscle dissatisfaction, and concerns about shoulder width (Cafri and Thompson, 2004). Rather, recent research, both on the general adolescent population and on T1D youths, suggested that gender-based patterns of body dissatisfaction can be identified (i.e., females have a desire for thinness, males prefer to be thinner or larger), and different corresponding DEBs could be recognized (i.e., excessive exercise vs. restraint activities for weight loss) (McCabe and Ricciardelli, 2004; Lawler and Nixon, 2011; d’Emden et al., 2013; Araia et al., 2017).

Also, in a recent study on adolescents with T1D and DEB, body image problems, as assessed by adopting a gender-specific self-report tool, were more frequently found in adolescents with T1D but no DEB (Troncone et al., 2019). However, in that study, due to the lack of a control group, no comparison of body image problems in adolescents with and without T1D was carried out.

The aims of the present study were: (a) to compare adolescents with diabetes and well-matched healthy peers regarding body image problems using a gender-specific tool, and (b) to analyze the relationship between body image problems and DEB in adolescent males and females with and without T1D.

Socio-demographic characteristics (age, SES) and clinical diabetes-related factors (BMI, Hb1Ac, and duration of illness) were also evaluated.

This study sought to address the following questions:

(1) Are there differences in body image problems between adolescents with T1D and without T1D?
(2) In adolescents with T1D and without T1D, is there a relationship between body image problems and DEBs?

In keeping with past research, the present study hypothesizes that in comparison to healthy peers, adolescents with T1D show significant differences in body image problems, and body image problems are expected to be significantly related to DEBs in both groups.

## Materials and Methods

### Participants

Participants were recruited from patients attending a southern Italy pediatric diabetes center from April to November 2018. To be included in the study, patients had to satisfy the following inclusion criteria: aged 13–18 years; T1D diagnosed from at least 1 year of age; and absence of any significant developmental, cognitive, psychological, or medical conditions. An examination of participants with T1D clinical records was systematically conducted to ascertain that the inclusion/exclusion criteria were met.

Control subjects were matched to the clinical group for age and gender; they were recruited from September 2018 to May 2019 from students attending schools in the same geographic area as the case. All children with known physical or psychological handicaps, as assessed by their teachers, were excluded from the control group.

### Measures

#### Socio-Demographic and Clinical Data

A brief interview schedule was designed *ad hoc* for the study and was performed by clinicians to record participants’ demographic and clinical data including age, sex, height, weight, (absence of) significant medical or psychological conditions (all participants), along with patients’ duration of illness and current HbA1c values (only participants with T1D).

##### Weight status

The BMI was used as a measure of actual weight status. Given that in children and adolescents this index varies based on age and gender, the BMI *z*-score was calculated for each subject based on gender, age, weight, and height according to the Center for Disease Control (CDC) growth curve tables (Kuczmarski et al., 2000).

##### Socioeconomic status

The socio-economic level assessment (SES) was carried out using the Barratt Simplified Measure of Social Status (BSMSS), based on information provided by parents (Barratt, 2006). The index is calculated as the combination of the level of schooling and the work of both parents. The SES value varies from 8 (indicating that neither parent has finished lower secondary school, and the parents have low-level employment) to 66 (both parents have obtained a post degree and carry out high-level occupations), where higher scores indicate higher socioeconomic levels. The scores categorize participants into five different SES groups: 55–66 = I; 40–54 = II; 30–39 = III; 20–29 = IV; 8–19 = V.

#### Disordered Eating Behaviors (DEBs)

To identify adolescents with DEBs, both a diabetes specific measure and a generic tool were adopted.

Diabetes Eating Problems Survey revised (DEPS-r) is a self-report questionnaire specifically designed to screen DEB in individuals with T1D (Markowitz et al., 2010). It was also developed to assess weight-reducing tools used by T1D patients, such as insulin omission or manipulation, that a generic DEB tool usually may fail to identify. It consists of 16 items whose answers are scored on a six-point Likert scale (ranging from 0 = never to 5 = always, in relation to frequency of the behavior). In line with previous research, scores equal to or higher than 20 were considered as suggesting high risk for DEB (Saßmann et al., 2015; Wisting et al., 2018, 2019). The DEPS-r is the most widely validated DEB tool for adolescents with T1D, and it has been demonstrated to possess high internal reliability and high concurrent/criterion validity when compared to a gold standard clinical interview; it also has convergent validity with significant correlations with other validated measures of eating disorders (i.e., EAT-12, EDI, SCOFF, and EDE-Q) (Pursey et al., 2020). A validated Italian version of the DEPS-r was used in this study (Pinna et al., 2017).

Eating Disorder Inventory – 3 Referral Form (EDI-3RF) is a self-report questionnaire intended to assess the presence of at-risk symptoms for developing DEB. It is a 25-item abbreviated form of the EDI-3 (Giannini and Conti, 2008) and includes three scales: (1) drive for thinness (DT), composed of seven items evaluating excessive concern for diet and fear of gaining weight; (2) bulimia (B), composed of eight items assessing preoccupation and tendency toward episodes of overfeeding; and (3) body dissatisfaction (BD), composed of 10 items measuring dissatisfaction with body shape and with specific body areas that tend to worry people suffering from eating disorders (i.e., the stomach, hips, thighs, buttocks, etc.). Respondents are asked to rate their agreement with each statement on a six-point Likert scale (always, usually, often, sometimes, rarely, and never). Higher scores are associated with a greater degree of DEB. The EDI-3RF has shown good psychometric properties for all three subscales (Garner, 2004). The present study used the Italian version of the EDI-3RF translated and validated by Giannini and Conti (2008). Since BD items focused on more female-centric thinness concerns, body dissatisfaction data collected by the EDI-3RF scale were not considered to accurately gauge body image problems in the present study.

#### Body Image Problems

According to evidence supporting the crucial role played by sociocultural influences on body dissatisfaction (Knauss et al., 2007; Stefanile et al., 2009; Girard et al., 2018), body image problems were conceptualized as appearance-ideal internalization and appearance pressures and assessed using the Sociocultural Attitudes Toward Appearance Questionnaire-4 (SATAQ-4R) (Schaefer et al., 2015). Internalization of the appearance ideal can be considered to be the degree of endorsement of prevailing cultural ideal standards of appearance (e.g., thinness for females and muscularity for males) as one’s own personal appearance standard (Thompson and Stice, 2001) and perceived sociocultural appearance-related influences such as the pressure to modify one’s physical appearance in order to reach such ideals (Thompson et al., 2004). SATAQ-4R is a gender-specific self-report tool composed of 31 items in the female version and 28 items in the male version, both including seven scales. Four scales evaluate the perceived pressure from family, peers, significant others, and the media to improve one’s appearance and attain a thin (SATAQ-4R-Female) or muscular (SATAQ-4R-Male) body. The remaining three sub-scales evaluate the desire to attain a lean body, Internalization of Thin/Low body fat; to attain a muscular physique, Internalization of Muscular athletic physical ideal; and overall concern with personal appearance, Internalization of General Attractiveness.

Respondents are asked to rate their agreement with each statement on a five-point Likert scale with response options from 1 (definitely disagree) to 5 (definitely agree). Higher scores indicate greater internalization and adhesion to the ideal aspect proposed by the current cultural influences and related perceived pressures. According to the most recent validation data, the SATAQ-4R scales showed excellent reliability and good validity converging with other body image measurements (Schaefer et al., 2015). Similarly, the Italian SATAQ-4R validation data provided evidence for the validity and reliability of the male and female versions (Stefanile et al., 2019).

### Procedure

Clinical sample data were collected when patients were waiting for routine diabetes visits; data for the control group were collected when students were at school without the presence of a teacher. If children were absent on testing days, a new evaluation was scheduled. Eligible participants were given a written information form describing the project and requesting parental and participant consent. An oral explanation of what they were agreeing to do was provided as well. Those who agreed to participate were enrolled in the study after the written informed consents were obtained. Participants were not compensated for participating in the study nor for completing the questionnaires.

Test administrations were carried out individually, anonymously, in a quiet and comfortable room made available by the service/school and performed by two psychologists adequately trained in techniques and supervised by the coordinator of the research group. The questionnaires took about 20 min to complete. Once questionnaires were completed, measurements of the actual height and weight of participants were made.

The study was approved by the local Ethics Committee (n. 355) and was performed according to the principles of the Helsinki Declaration II.

### Statistical Analysis

To assess the homogeneity of the scale, Cronbach’s alpha (α) was computed. Chi-square testing was used to test frequencies between groups and Student’s *t*-tests to compare means of continuous variables between two groups (i.e., male/female and patients/control). For SATAQ-4R, due to the non-overlapping number of items in the male/female version sub-scales, it was not possible to make comparisons between male and female scores.

Hierarchical multiple regression analyses, with three blocks entered, were conducted to evaluate the relationship between DEB and body image problems. Age and SES were entered in Step 1 as control variables; zBMI along with disease factors including illness duration and HbA1c (for participants with T1D) were entered in Step 2; SATAQ-4R scores were added in the last step of the regression. The DEPS-r scores (only for participants with T1D), DT, and Bulimia EDI-3RF scores were the dependent variables. To analyze whether this relationship interacted with gender or illness, regression analyses were performed separately for males and females, for participants with T1D, and for the controls. Tolerance values of >0.1 were considered acceptable to exclude multicollinearity (Cohen et al., 2003).

Results were considered significant at alpha = 0.05. The statistical analyses were performed with the Statistical Package for the Social Sciences (SPSS), version 21.0 for Macintosh.

## Results

### Sample Characteristics

Of all adolescents with T1D who were asked to be included in the study (*N* = 261), 23.4% refused to consent to participation (*N* = 61). For the control group, of all students invited (*N* = 365), 23.01% of them refused (*N* = 84). Then, the students whose parents provided written consent (*N* = 281) and who were best age- and gender-matched to the clinical sample subjects were selected to be evaluated (*N* = 183). Data on reasons for refusal were not formally collected, but parents’ worries that their children would undergo psychological evaluation and loss of the consent form by the child (especially for controls) at the time of evaluation were the most frequent reasons preventing participation.

The study sample consisted of 183 adolescents with T1D (98 girls, 85 boys) and 183 matched healthy peers.

The socio-demographic, anthropometric, and clinical characteristics of adolescents with T1D and controls, grouped by gender and means comparison, are reported in Table 1.

**TABLE 1:** Socio-demographic, anthropometric, and clinical characteristics in total sample, in female and male participants with and without T1D, and comparisons of means on the basis of gender and illness.

	**T1D**			**Controls**							
	**Total *N* = 183**	**Males *N* = 85**	**Females *N* = 98**	**Total *N* = 183**	**Males *N* = 85**	**Females *N* = 98**	**T1D m vs. T1D f**	**T1D vs. Ctrl**	**T1D m vs. Ctrl m**	**T1D f vs. Ctrl f**	**Ctrl m vs. Ctrl f**
											
	***M (SD)***	***M (SD)***	***M (SD)***	***M (SD)***	***M (SD)***	***M (SD)***	***p***	***p***	***p***	***p***	***p***
Age: years,	15.29 (1.39)	15.29 (1.33)	15.29 (1.45)	15.35 (1.36)	15.37 (1.28)	15.32 (1.44)	0.978	0.714	0.699	0.880	0.797
month (range)	(13.02–18.05)	(13.07–18.05)	(13.02–18)	(13.05–18.06)	(13.09–18.06)	(13.05–18.04)					
Hb1Ac %	7.94 (1.47)	7.77 (1.15)	8.09 (1.69)	–	–	–	0.145	–	–	–	–
Duration of illness: years, month	7.16 (3.68)	7.64 (3.99)	6.74 (3.34)	–	–	–	0.095	–	–	–	–
z-BMI	0.51 (0.79)	0.30 (0.82)	0.7 (0.72)	0.28 (0.97)	0.53 (0.84)	0.07 (1.03)	0.001	0.013	0.077	0.000	0.001
SES	28.01 (11.28)	28.79 (11.21)	27.32 (11.35)	32.31 (11.31)	33.07 (12.39)	31.66 (10.31)	0.382	0.000	0.020	0.006	0.407

*Data are presented as mean values and standard deviations unless otherwise stated. T1D, type 1 diabetes; N, number of subjects; HbA1c, glycated hemoglobin; z-BMI, standardized body mass index; SES, socioeconomic status; m, males; f, females.*

There were no statistically significant differences between the T1D group and the control group in terms of distribution of sex and age, nor were there statistically significant differences between girls and boys with T1D in terms of age, SES, glycemic control, and duration of illness (Table 1). Compared to control participants, adolescents with T1D had higher zBMI [*t*(364) = 2.506, *p* = 0.013], especially girls [zBMI *t*(194) = 4.956, *p* < 0.0001], and lower SES {total sample [*t*(364) = −3.620, *p* < 0.0001]; boys *t*(168) = −2.342, *p* = 0.020; girls *t*(194) = −2.784, *p* = 0.006)}. In participants with T1D, girls reported higher zBMI values than boys [*t*(181) = −3.463, *p* = 0.001], while in the control group, boys had higher zBMI than girls [*t*(181) = 3.281, *p* = 0.001] (Table 1).

### Disordered Eating Behaviors (DEBs)

Table 2 shows DEPS-r and EDI-3RF mean scores (SD) in the total sample, male and female adolescents with and without T1D, frequency of DEBs, insulin misuse, and means comparison on the basis of gender and illness.

**TABLE 2:** DEPS-r and EDI-3RF Cronbach’s alpha coefficients, mean scores in total sample, male and female adolescents with and without T1D.

		**T1D**			**Controls**							
** Scale**		**Total *N* = 183**	**Males *N* = 85**	**Females *N* = 98**	**Total *N* = 183**	**Males *N* = 85**	**Females *N* = 98**	**T1D m vs. T1D f**	**T1D vs. Ctrl**	**T1D m vs. Ctrl m**	**T1D f vs. Ctrl f**	**Ctrl m vs. Ctrl f**
												
	**α**	***M (SD)***	***M (SD)***	***M (SD)***	***M (SD)***	***M (SD)***	***M (SD)***	***p***	***p***	***p***	***p***	***p***
DEPS-r	0.852	19.4 (12.92)	15.88 (10.18)	22.46 (14.25)	–	–	–	0.001	–	–	–	–
Score ≥ 20% (N)	–	37.7 (69)	12.6 (23)	25.1 (46)	–	–	–	0.006	–	–	–	–
Int. ins. red % (N)^†^	–	61.7 (113)	28.9 (53)	32.8 (60)	–	–	–	0.876	–	–	–	–
Int. ins. om. % (N)^†^	–	20.22 (37)	9.3 (17)	10.9 (20)	–	–	–	0.945	–	–	–	–
EDI-3RF												
Drive for thinness	0.872	14.23 (9.75)	8.96 (7.53)	18.81 (9.154)	9.19 (7.61)	5.8 (5.64)	12.13 (7.89)	0.000	0.000	0.002	0.000	0.000
Bulimia	0.830	6.89 (7.97)	4.78 (5.12)	8.71 (9.44)	4.13 (4.43)	3.34 (3.77)	4.81 (4.85)	0.001	0.000	0.039	0.000	0.025
Body diss.	0.742	16.67 (7.98)	14.15 (7.97)	18.86 (7.35)	12.91 (9.03)	9.79 (7.22)	15.62 (9.59)	0.000	0.000	0.000	0.009	0.000

*Frequency of DEBs and insulin misuse as measured by DEPS-r in total sample, males and females with T1D. Comparisons of means on the basis of gender and illness. Data are presented as mean values and standard deviations unless otherwise stated. ^†^Answers to DEPS-r item 2 and item 8 (from “rarely” to “often”) indicating the presence of intentional insulin omission/reduction actually or in the last 6 months. T1D, type 1 diabetes; N, number of subjects; DEPS-r, iabetes Eating Problems Survey-revised; Int. ins. red, intentional insulin reduction; Int. ins. om., intentional insulin omission; EDI-3RF, Eating Disorder Inventory-3 Referral Form; Body diss., body dissatisfaction; m, males; f, females.*

DEPS-r as well as all scales of the EDI-3RF demonstrated good internal consistency. Participants with T1D showed diabetes-specific eating problems and insulin misuse; among these participants, girls reported higher DEPS-r mean scores than boys [*t*(181) = −3.452, *p* = 0.001] and more frequently showed DEBs than boys (*X*^2^ = 7.659, *p* = 0.006). No differences between males and females with T1D were observed in insulin reduction or omission (Table 2).

Participants with T1D reported higher scores than controls in all EDI-3RF [total sample *t*(364) = 5.517, *p* < 0.0001; DT *t*(364) = 5.517, *p* < 0.0001; Bulimia *t*(364) = 4.094, *p* < 0.0001; BD *t*(364) = 4.219, *p* < 0.0001] also when direct comparison was carried out between males with and without T1D [DT *t*(168) = 3.101, *p* = 0.002; Bulimia *t*(168) = 2.081, *p* = 0.039; BD *t*(168) = 3.741, *p* < 0.0001] and between females with and without T1D [DT *t*(194) = 5.466, *p* < 0.0001; Bulimia *t*(194) = 3.644, *p* < 0.0001; BD *t*(194) = 2.649, *p* = 0.009]. Females showed higher EDI-3RF subscale scores than males both in patients [DT *t*(181) = −7.867, *p* < 0.0001; Bulimia *t*(181) = −3.431, *p* = 0.001; BD *t*(181) = −4.150, *p* < 0.0001] and in controls [DT *t*(181) = −6.158, *p* < 0.0001; Bulimia *t*(181) = −2.255, *p* = 0.025; BD *t*(181) = −4.591, *p* < 0.0001] (Table 2).

### Body Image Problems

In Table 3 mean (SD) of male and female SATAQ-4R scores are shown along with a T1D vs. control comparison.

**TABLE 3:** SATAQ-4R Cronbach’s alpha coefficients, mean scores in male and female adolescents with T1D and without T1D.

		**Males**			**Females**		
**Scale**		**T1D *N* = 85**	**Controls *N* = 85**	**T1D m vs. Ctrl m**	**T1D *N* = 98**	**Controls *N* = 98**	**T1D f vs. Ctrl f**
	**α**	***M (SD)***	***M (SD)***	***p***	***M (SD)***	***M (SD)***	***p***
SATAQ-4R^†^	m/f						
Int. thin/low body	0.811/0.768	4.59 (2.54)	4.81 (1.87)	0.515	12.89 (4.64)	12.78 (3.77)	0.853
Int. muscular	0.883/0.583	11.12 (4.37)	11.46 (3.49)	0.574	10.92 (4.28)	10.68 (3.17)	0.663
Int. general attract	0.743/0.776	7.46 (2.07)	7.48 (2.08)	0.941	22.56 (5.63)	23.8 (3.98)	0.078
Press. family	0.799/0.800	9.32 (4.39)	9.64 (3.88)	0.618	9.81 (4.6)	8.46 (3.7)	0.025
Press. peers	0.901/0.869	7.21 (3.99)	8.25 (3.95)	0.091	7.02 (4.02)	6.54 (3.61)	0.381
Press. sign. others	0.868/0.882	9.15 (4.93)	9.34 (4.11)	0.787	7.85 (4.35)	6.86 (3.65)	0.086
Press. media	0.899/0.950	9.89 (5.46)	10.41 (4.56)	0.503	8.76 (5.5)	10.52 (5.2)	0.022

*Comparisons of means on the basis of gender (m vs. m; f vs. f) and illness. Data are presented as mean values and standard deviations unless otherwise stated. ^†^For SATAQ-4R scores, Cronbach’s alpha of SATAQ-4R was reported as male/female values. T1D, type 1 diabetes; N, number of subjects; SATAQ-4R, Sociocultural Attitudes Toward Appearance Questionnaire-4-Revised; Int. thin/low body, internalization of the thin-low body fat physical ideal; Int. muscular, internalization of the muscular-athletic physical ideal; Int. general attract, internalization of a more general desire to be attractive or in good shape; Press. family, pressure from family; Press. Peers, pressure from peers; Press. sign. others, pressure from significant others; Press. media, pressure from media; m, males; f, females; Ctrl, controls.*

All scales of the SATAQ-4R (male and female versions) demonstrated good internal consistency, except for lower values of Internalization of Muscular athletic physical ideal – female version.

No differences were found between male adolescents with T1D and male controls regarding body image problems or between females with T1D and controls (Table 3). Females with T1D showed higher scores than females of the control group in Pressure from Family [*t*(194) = 2.258, *p* = 0.025] and lower scores than healthy females peers in Pressure from Media [*t*(194) = −2.308, *p* = 0.022] (Table 3).

### Body Image Problems and DEBs

In all regression analyses, the collinearity statistics of the predictors showed tolerance values ranging from 0.290 to 0.996, thus excluding multicollinearity (Tables 4–6).

**TABLE 4:** Summary of linear regression analyses of variables predicting participants with T1D’ DEBs (DEPS-r score) in male and female adolescents with T1D.

	**T1D Males (*N* = 85)**					**TD1 Females (*N* = 98)**				
**Variables**	***B***	***SE B***	**β**	***p***	**Collinearity tolerance**	***B***	***SE B***	**β**	***p***	**Collinearity tolerance**
**Step 1**										
Age	−0.135	0.789	−0.018	0.866	0.988	1.315	0.988	0.135	0.187	0.996
SES	−0.310	0.095	−0.342	0.002	0.988	−0.179	0.127	−0.143	0.162	0.996
**Step 2**										
Age	−0.356	0.798	−0.047	0.657	0.884	1.039	1.005	0.106	0.304	0.880
SES	−0.257	0.095	−0.283	0.008	0.882	−0.124	0.123	−0.099	0.314	0.971
z-BMI	4.001	1.249	0.321	0.002	0.959	4.557	1.963	0.231	0.022	0.944
Duration of illness	0.100	0.273	0.039	0.714	0.844	−0.179	0.442	−0.042	0.687	0.868
HbA1c	1.758	0.940	0.198	0.065	0.857	1.749	0.829	0.210	0.038	0.945
**Step 3**										
Age	−0.248	0.640	−0.032	0.700	0.820	0.688	0.850	0.070	0.420	0.862
SES	−0.198	0.075	−0.218	0.010	0.853	−0.180	0.109	−0.144	0.103	0.860
z-BMI	1.092	1.076	0.088	0.314	0.770	−1.136	1.890	−0.057	0.549	0.714
Duration illness	0.557	0.222	0.218	0.014	0.762	−0.133	0.401	−0.031	0.741	0.741
HbA1c	1.021	0.775	0.115	0.192	0.751	0.994	0.715	0.119	0.168	0.889
**SATAQ-4R**										
Int. thin/low body	1.489	0.350	0.372	0.000	0.754	0.473	0.335	0.152	0.161	0.561
Int. muscular	0.309	0.222	0.133	0.168	0.633	−0.290	0.331	−0.087	0.383	0.656
Int. general attract	0.299	0.407	0.061	0.465	0.838	−0.058	0.245	−0.023	0.812	0.708
Press. family	−0.260	0.326	−0.112	0.427	0.290	0.769	0.428	0.248	0.076	0.341
Press. peers	−0.035	0.333	0.014	0.917	0.335	−0.112	0.433	−0.032	0.797	0.433
Press. sign. others	0.862	0.251	0.418	0.001	0.388	0.875	0.452	0.268	0.056	0.340
Press. media	0.170	0.252	0.091	0.502	0.315	0.628	0.282	0.243	0.029	0.546

*T1D, type 1 diabetes; SES, socioeconomic status; z-BMI, standardized body mass index; HbA1c, glycated hemoglobin; SATAQ-4R, Sociocultural Attitudes Toward Appearance Questionnaire-4-Revised; Int. thin/low body, internalization of the thin-low body fat physical ideal; Int. muscular, internalization of the muscular-athletic physical ideal; Int. general attract., internalization of a more general desire to be attractive or in good shape; Press. family, pressure from family; Press. Peers, pressure from peers; Press. sign. others, pressure from significant others; Press. media, pressure from media. TD1 Male: R^2^ = 0.116 for Step 1 (p = 0.006); ΔR^2^ = 0.123 for Step 2 (p = 0.001); ΔR^2^ = 0.348 for Step 3 (p < 0.001), tot R^2^ = 0.686. TD1 Female: R^2^ = 0.041 for Step 1 (p = 0.141); ΔR^2^ = 0.111 for Step 2 (p = 0.009); ΔR^2^ = 0.299 for Step 3 (p < 0.001), tot R^2^ = 0.451.*

**TABLE 5:** Summary of linear regression analyses of variables predicting participants’ DEBs (EDI-3RF Drive for Thinness score) in male and female adolescents with T1D and without T1D.

	**T1D**										**Controls**									
**Variables**	**Males *N* = 85**					**Females *N* = 98**					**Males *N* = 85**					**Females *N* = 98**				
	***B***	***SE B***	**β**	***p***	**Collinearity tolerance**	***B***	***SE B***	**β**	***p***	**Collinearity tolerance**	***B***	***SE B***	**β**	***p***	**Collinearity tolerance**	***B***	***SE B***	**β**	***p***	**Collinearity tolerance**
**Step 1**																				
Age	−0.406	0.626	−0.072	0.519	0.988	0.811	0.646	0.129	0.212	0.996	0.388	0.501	0.087	0.441	0.988	0.111	0.577	0.020	0.848	0.989
SES	−0.043	0.074	−0.064	0.563	0.988	0.038	0.083	0.047	0.648	0.996	0.003	0.052	0.006	0.959	0.988	−0.008	0.080	−0.010	0.920	0.989
**Step 2**																				
Age	−0.259	0.625	−0.046	0.680	0.884	0.584	0.647	0.093	0.370	0.880	0.443	0.456	0.100	0.334	0.987	0.353	0.579	0.063	0.544	0.948
SES	0.021	0.074	−0.031	0.780	0.882	0.070	0.079	0.087	0.375	0.971	−0.003	0.048	−0.007	0.949	0.987	0.003	0.079	0.003	0.974	0.985
Duration illness	−0.266	0.214	−0.141	0.217	0.959	0.015	0.285	0.006	0.957	0.944										
z-BMI	20.933	0.978	0.318	0.004	0.844	4.829	1.264	0.379	0.000	0.868	2861	0.686	0.426	0.000	0.998	1.647	0.801	0.213	0.043	0.957
HbA1c	1.153	0.736	0.176	0.121	0.857	−0.098	0.534	−0.018	0.855	0.945										
**Step 3**																				
Age	−0.074	0.549	−0.013	0.893	0.820	0.189	0.513	0.030	0.714	0.862	0.011	0.432	0.002	0.980	0.907	−0.285	0.500	−0.051	0.571	0.709
SES	0.022	0.064	0.033	0.729	0.853	−0.016	0.066	−0.020	0.806	0.860	0.024	0.047	0.051	0.619	0.820	−0.098	0.065	−0.128	0.137	0.794
Duration illness	0.047	0.190	0.025	0.807	0.770	0.242	0.242	0.088	0.319	0.714										
z-BMI	0.717	0.923	0.078	0.440	0.762	1.367	1.141	0.107	0.234	0.741	2.166	0.766	0.323	0.006	0.657	0.883	0.703	0.108	0.239	0.693
HbA1c	0.598	0.665	0.091	0.372	0.751	−0.581	0.432	−0.108	0.182	0.889										
**SATAQ-4R**																				
Int. thin/low body	1.091	0.300	0.368	0.001	0.754	0.727	0.202	0.363	0.001	0.561	0.861	0.325	0.285	0.010	0.736	1.125	0.205	0.537	0.000	0.598
Int. muscular	0.023	0.190	0.013	0.904	0.633	0.178	0.200	0.083	0.376	0.656	−0.088	0.175	−0.055	0.615	0.715	−0.303	0.215	−0.121	0.162	0.776
Int. general attract	0.256	0.349	0.071	0.465	0.838	−0.062	0.148	−0.037	0.678	0.708	0.724	0.277	0.266	0.011	0.828	0.069	0.183	0.034	0.707	0.689
Press. family	−0.155	0.280	−0.090	0.582	0.290	0.145	0.259	0.072	0.578	0.341	−0.248	0.215	−0.170	0.251	0.395	0.402	0.244	0.187	0.104	0.442
Press. peers	−0.084	0.286	−0.045	0.768	0.336	−0.164	0.261	−0.072	0.531	0.433	0.223	0.218	0.158	0.311	0.356	−0.208	0.273	−0.095	0.449	0.369
Press. sign. others	0.580	0.215	0.380	0.009	0.388	0.235	0.273	0.112	0.392	0.340	0.191	0.209	0.140	0.363	0.368	0.195	0.254	0.090	0.446	0.417
Press. media	0.262	0.216	0.190	0.230	3.15	0.577	0.170	0.347	0.001	0.546	0.101	0.167	0.082	0.548	0.465	0.482	0.151	0.317	0.002	0.581

*TD1, type 1 diabetes; SES, socioeconomic status; z-BMI, standardized body mass index; HbA1c, glycated hemoglobin; SATAQ-4R, Sociocultural Attitudes Toward Appearance Questionnaire-4-Revised; Int. thin/low body, internalization of the thin-low body fat physical ideal; Int. muscular, internalization of the muscular-athletic physical ideal; Int. general attract., internalization of a more general desire to be attractive or in good shape; Press. family, pressure from family; Press. Peers, pressure from peers; Press. sign. others, pressure from significant others; Press. media, pressure from media. TD1 Male: R^2^ = 0.008 for Step 1 (p = 0.711); ΔR^2^ = 0.139 for Step 2 (p = 0.025); ΔR^2^ = 0.296 for Step 3 (p < 0.001) tot R^2^ = 0.444. TD1 Female: R^2^ = 0.018 for Step 1 (p = 0.426); ΔR^2^ = 0.138 for Step 2 (p = 0.008); ΔR^2^ = 0.364 for Step 3 (p = 0.000); tot R^2^ = 0.520. Control Male: R^2^ = 0.008 for Step 1 (p = 0.742); ΔR^2^ = 0.181 for Step 2 (p = 0.001); ΔR^2^ = 0.204 for Step 3 (p = 0.000); tot R^2^ = 0.393. Control Female: R^2^ = 0.001 for Step 1 (p = 0.975); ΔR^2^ = 0.043 for Step 2 (p = 0.240); ΔR^2^ = 0.463 for Step 3 (p = 0.000); tot R^2^ = 0.507.*

**TABLE 6:** Summary of linear regression analyses of variables predicting participants’ DEBs (EDI-3RF Bulimia score) in male and female adolescents with and without T1D.

	**T1D**										**Controls**									
**Variables**	**Males *N* = 85**					**Females *N* = 98**					**Males *N* = 85**					**Females *N* = 98**				
	***B***	***SE B***	**β**	***p***	**Collinearity tolerance**	***B***	***SE B***	**β**	***p***	**Colinearity tolerance**	***B***	***SE B***	**β**	***p***	**Collinearity tolerance**	***B***	***SE B***	**β**	***p***	**Collinearity tolerance**
**Step 1**																				
Age	0.490	0.423	0.128	0.250	0.988	0.216	0.659	0.033	0.743	0.996	0.483	0.332	0.161	0.150	0.988	−0.006	0.353	−0.002	0.987	0.989
SES	0.011	0.050	0.024	0.826	0.988	−0.162	0.084	−0.195	0.058	0.996	0.046	0.035	0.147	0.189	0.988	−0.034	0.049	−0.072	0.491	0.989
**Step 2**																				
Age	0.567	0.446	0.147	0.208	0.842	0.084	0.685	0.013	0.902	0.880	0.478	0.334	0.159	0.156	0.987	0.183	0.350	0.054	0.601	0.948
SES	0.031	0.053	0.068	0.561	0.882	−0.133	0.084	−0.159	0.116	0.971	0.047	0.035	0.149	0.185	0.987	−0.026	0.048	−0.054	0.591	0.985
Duration illness	−0.110	0.152	−0.086	0.471	0.959	−0.086	0.301	−0.030	0.776	0.944										
z-BMI	0.808	0.697	0.129	0.250	0.844	3.398	1.338	0.258	0.013	0.868	−0.285	0.501	−0.063	0.572	0.998	1.286	0.484	0.271	0.009	0.957
HbA1c	0.769	0.525	0.173	0.147	0.857	0.322	0.565	0.058	0.570	0.945										
**Step 3**																				
Age	0.459	0.437	0.120	0.297	0.820	−0.134	0.549	−0.021	0.808	0.862	0.464	0.341	0.155	0.178	0.907	−0.012	0.359	−0.003	0.974	0.709
SES	0.060	0.051	0.132	0.240	0.853	−0.163	0.070	−0.196	0.023	0.860	0.071	0.037	0.227	0.062	0.820	−0.008	0.047	−0.017	0.865	0.794
Duration illness	0.063	0.151	0.049	0.681	0.770	−0.090	0.259	−0.032	0.729	0.714										
z-BMI	0.250	0.736	0.040	0.735	0.762	−0.253	1.221	−0.018	0.848	0.741	−0.163	0.606	−0.036	0.789	0.657	0.945	0.505	0.199	0.065	0.693
HbA1c	0.385	0.530	0.086	0.470	0.751	−0.238	0.462	−0.043	0.608	0.889										
**SATAQ-4R**																				
Int. thin/low body	0.232	0.239	0.115	0.335	0.754	0.042	0.217	0.020	0.847	0.561	0.015	0.257	0.007	0.953	0.736	0.115	0.147	0.090	0.436	0.598
Int. muscular	−0.004	0.152	−0.003	0.980	0.633	−0.250	0.214	−0.113	0.246	0.656	0.227	0.139	0.210	0.105	0.715	−0.020	0.154	−0.013	0.897	0.776
Int. general attract	−0.065	0.278	−0.026	0.816	0.838	−0.213	0.158	−0.125	0.182	0.708	−0.315	0.219	−0.171	0.156	0.828	0.053	0.131	0.043	0.686	0.689
Press. family	−0.259	0.223	−0.222	0.250	0.290	0.372	0.277	0.180	0.182	0.341	0.056	0.170	0.057	0.744	0.395	0.015	0.175	0.012	0.931	0.442
Press. peers	0.323	0.228	0.252	0.160	0.336	−0.014	0.280	−0.006	0.961	0.433	0.155	0.173	0.163	0.372	0.356	0.769	0.196	0.572	0.000	0.369
Press. sign. others	0.307	0.172	0.296	0.078	0.388	0.659	0.292	0.303	0.027	0.340	−0.019	0.165	−0.021	0.907	0.368	−0.224	0.182	−0.168	0.222	0.417
Press. media	0.093	0.172	0.099	0.592	0.315	0.730	0.182	0.425	0.000	0.546	0.040	0.132	0.048	0.765	0.465	0.033	0.109	0.035	0.765	0.581

*T1D, type 1 diabetes; SES, socioeconomic status; z-BMI, standardized body mass index; HbA1c, glycated hemoglobin; SATAQ-4R, Sociocultural Attitudes Toward Appearance Questionnaire-4-Revised; Int. thin/low body, internalization of the thin-low body fat physical ideal; Int. muscular, internalization of the muscular-athletic physical ideal; Int. general attract., internalization of a more general desire to be attractive or in good shape; Press. Family, pressure from family; Press. Peers, pressure from peers; Press. sign. Others, pressure from significant others; Press. Media, pressure from media. T1D Male: R^2^ = 0.016 for Step 1 (p = 0.512); ΔR^2^ = 0.043 for Step 2 (p = 0.424); ΔR^2^ = 0.175 for Step 3 (p = 0.058); tot R^2^ = 0.235. T1D Female: R^2^ = 0.040 for Step 1 (p = 0.148); ΔR^2^ = 0.074 for Step 2 (p = 0.047); ΔR^2^ = 0.370 for Step 3 (p = 0.000); tot R^2^ = 0.485. Control Male: R^2^ = 0.042 for Step 1 (p = 0.181); ΔR^2^ = 0.004 for Step 2 (p = 0.293); ΔR^2^ = 0.119 for Step 3 (p = 0.194); tot R^2^ = 0.166. Control Females: R^2^ = 0.005 for Step 1 (p = 0.786); ΔR^2^ = 0.070 for Step 2 (p = 0.063); ΔR^2^ = 0.252 for Step 3 (p = 0.000); tot R^2^ = 0.327.*

#### DEBs as Diabetes-Specific Eating Problems

Table 4 presents the results of a hierarchical regression predicting DEB (DEPS-r scores) in male and female adolescents with T1D.

The entire set of variables explained a significant amount of variance in both girls’ and boys’ DEPS-r scores. The variables entered in the first step did not contribute to DEB (all *p* > 0.05), except for SES values found negatively associated to DEPS-r scores in male participants. The clinical disease variables entered in the second step significantly increased *R*^2^ in both genders. In particular, zBMI was found to be a significant predictor of DEPS-r scores in all patients as well as HbA1c in female patients. In the third step, body image problems significantly increased DEPS-r score variance in both genders, indicating the role of Internalization of Thin/Low body fat and appearance Pressure from Significant Others, along with illness duration for males with T1D and of appearance Pressure from Media for females (Table 4).

#### DEB as Drive for Thinness

Table 5 presents the results of a hierarchical regression predicting DEB (DT EDI-3RF scores) in male and female adolescents with and without T1D.

The entire set of variables explained a significant amount of variance in the DT scores of both T1D girls and boys, ‘as well as in control-group girls’ and boys’ DT scores.

In adolescents with and without T1D, the variables entered in the first step were not significantly associated with DEB (all *p* > 0.05). The clinical disease variables entered in the second step significantly increased *R*^2^ in males and females with T1D and in males of the control group, but not in females. In particular, zBMI values were positively and significantly associated with DT both in patients and in controls. The third step added the body image measure, with a resulting final *R*^2^ > 0.393 for all groups and a significant change in all *R*^2^. Internalization of Thin/Low body fat scores were found significantly associated to DT scores in all groups. Internalization of General Attractiveness was found associated to DEBs in males of the control group, while Pressure from Significant Others in males with T1D and Pressure from Media in females with and without T1D were significantly associated with DT scores (Table 5).

#### DEB as Bulimia

Table 6 presents the results of a hierarchical regression predicting DEB (Bulimia EDI-3RF scores) in male and female adolescents with and without T1D.

The entire set of variables explained a significant amount of variance in the bulimia scores of both T1D girls and boys, ‘as well as in control-group girls’ and boys’ bulimia scores. In the first step, age and SES did not account for significant variance in bulimia scores for all groups (all *p* > 0.05). In the second step, the clinical variables contributed significantly to the regression only for females, with a resulting significant change in *R*^2^ for T1D and for controls. In particular, zBMI values were found significantly associated with bulimia scores in adolescent females with and without T1D. The third step added the body image problem measures, with a resulting change in *R*^2^ only for females with T1D and of control. Pressure from Significant Others and from Media were significantly associated with bulimia scores in females with T1D; Pressure from Peers scores were significantly associated with bulimia scores in healthy females (Table 6).

## Discussion

This is the first study aimed at investigating and comparing body image problems in adolescents with T1D and a well-matched control group using a gender-specific tool and evaluating associations between such body image problems and DEB in both groups. For participants with T1D, it also explored the prevalence of DEBs and related behaviors of insulin manipulation as a weight loss/control strategy.

Type 1 diabetes and healthy groups differed very little in socio-demographic characteristics. Participants with T1D, especially females, reported zBMI values significantly higher than healthy peers. Additionally, in the T1D group but not in the controls, females had zBMI higher than males.

### Disordered Eating Behaviors (DEBs)

As confirmed by both eating problem measures adopted in this study (i.e., diabetes-specific measure and generic tool) and in line with previous evidence from Italian samples (Pinna et al., 2017; Cherubini et al., 2018; Troncone et al., 2019), DEBs were more frequent in adolescents with T1D than in healthy peers. DEBs were found in both genders, although - as reported in DEB studies with adolescents from the general and the T1D population (Neumark-Sztainer et al., 2011; Wisting et al., 2013; Baechle et al., 2014; Colton et al., 2015) – they were found to a greater extent in girls than in boys, regardless of health status.

It is worth noting that measuring DEB through self-reporting tools adapted for use with T1D patients allowed this study to identify certain diabetes-specific eating behaviors (i.e., insulin omission/reduction, as found in the present sample) that a generic tool would surely overlook, with the additional risk of misidentifying appropriate diabetes management behaviors as DEBs (e.g., some generic tools ask whether a subject keeps a count of the food they have eaten, inflating DEB symptom scores, since counting carbohydrates is now standard in the management of T1D).

### Body Image Problems

When comparing body image problems, no substantial differences between patient and non-patient groups in the pattern of the gender-specific tool’s scores were observed.

Results from previous studies have been inconclusive as to whether adolescents with T1D are more likely to have body image problems compared with other adolescents. Several researchers found that youths with diabetes more frequently reported concerns and dissatisfaction about their bodies (Colton et al., 1999; Neumark-Sztainer et al., 2002; Kichler et al., 2008; Shaban, 2010; Kaminsky and Dewey, 2013; Philippi et al., 2013; Pinquart, 2013; Kaminsky and Dewey, 2014; Troncone et al., 2016, 2018). In contrast, and more in line with the findings of this study, other studies have found no significant differences in body image problems between adolescents with T1D and healthy peers (Striegel-Moore et al., 1992; Meltzer et al., 2001; Ackard et al., 2008; Kaminsky and Dewey, 2013; Falcão and Francisco, 2017). The use of different measures (silhouettes chart, self-report, one statement about satisfaction of his/her own weight, etc.) in assessing body image constructs that are differently defined (body dissatisfaction, negative body image, shape concerns etc.), not always taking into account gender-based patterns of body image concerns, could account for some of the inconsistency in the research results.

In the present study, the absence of significant differences between patients and controls in body image problems leads one to perceive body image problems more as an important adolescent developmental issue (consisting of the need to face the impact of body changes, giving more importance to their body image, putting more focus and energy into searching for acceptance by peers etc.) rather than as a result of the challenges of living with a chronic illness.

The negative effects of diabetes on body image seemed observable to a small extent only in females with T1D, where pressure from family and the media to maintain a thin body differed significantly compared to females without T1D.

Considering that adolescent females with T1D, as also found in this study, are often heavier than their non-diabetic peers as a side effect of intensified insulin therapy and other life-style factors (Domargård et al., 1999; Fröhlich-Reiterer et al., 2014; Minges et al., 2017), it could be hypothesized that their parents may further stress the importance of thinness and consequently pressure daughters to take control of their weight to better meet diabetes rules. Moreover, the demanding pressure to adhere to daily self-care activities required for T1D management could provide a possible explanation of why the pressure to be thin, generally exerted by the media on girls (Grabe et al., 2008; Scharrer, 2013), it was perceived more weakly by adolescents with T1D in our sample than by their healthy peers.

### Body Image Problems and DEBs

With respect to variables predicting DEB, collectively, the present results indicate that both in patients and in controls common predictors could be identified. In adolescents with and without T1D, zBMI and body image problems (both as appearance-ideal internalization and perceived sociocultural appearance) contributed to significantly increasing DEB risk. In particular, in all female participants, higher weight and body image problems predicted a drive for thinness and bulimic symptoms; in addition, in participants with T1D, HbA1c (whose values were analogous to population data in similar age groups, Clements et al., 2016; National Paediatric Diabetes Audit (NPDA), 2018) was found to contribute to diabetes eating problems, and SES was found to contribute to bulimic symptoms. Similarly, in all male participants, zBMI and body image problems predicted a drive for thinness; in those with T1D along these variables, SES and duration of illness predicted diabetes eating problems. None of the variables analyzed were found to significantly predict male bulimic symptoms, either in patients or controls.

Unsurprisingly, zBMI was found to play a role in predicting DEBs regardless of gender or illness, confirming that youths with higher weights have a higher prevalence of engaging in DEBs and unhealthy weight control practices, as indicated both in general and in the T1D adolescent population (Tanofsky-Kraff et al., 2004; Colton et al., 2007; Olmsted et al., 2008; Larson et al., 2009; Tse et al., 2012; Hanlan et al., 2013; Wisting et al., 2013). Generally, weight gain - most likely also related to the pubertal intensification of therapy (Bryden et al., 1999) – may lead to more intense dietary restraints and, consequently, to more frequent adoption of unhealthy strategies to lose weight (Stice, 2001).

Similarly, the association between DEBs and lower SES, poorer glycemic control, and duration of illness confirmed what has already been reported by previous studies on adolescents with T1D (Young et al., 2013; Pinhas-Hamiel et al., 2015; Cherubini et al., 2018; Troncone et al., 2019).

Overall, the current findings corroborate the existing research on general and T1D populations supporting the association between body image problems and DEBs (Colton et al., 1999; Striegel-Moore and Debra, 2002; Fairburn and Harrison, 2003; Striegel-Moore and Bulik, 2007; Kichler et al., 2008; Troncone et al., 2016, 2018; Amaral and Ferreira, 2017; Falcão and Francisco, 2017; Girard et al., 2018; Verbist and Condon, 2019; Araia et al., 2020).

On the basis of the significant associations between body image problems, weight, and DEB, one could speculate a vicious circle in which higher weight and body image problems may play a significant role in DEB development as well as adopting dysregulated eating habits that may favor, in turn, weight gain and body concerns increase (Field et al., 2003; Neumark-Sztainer et al., 2006). As a result, DEB can be further reinforced because weight gain may lead to higher levels of body dissatisfaction, more intense dietary restraint, and consequently more frequent adoption of unhealthy strategies to lose weight (Stice, 2001; Stice and Shaw, 2002).

In analyzing the relationship between weight, body image, and bulimic symptoms, gender differences were observed: body image problems, despite being evaluated taking into account gender-based body image concerns, were found to predict (along with weight) bulimic symptoms in females, but no such significant associations were found in males, regardless of presence of illness.

The literature exploring the main factors contributing to bulimic symptoms in youth with T1D and their association with gender is mixed: some evidence did not describe different bulimic symptom predictors in males and females (Kaminsky and Dewey, 2013) or constantly confirmed the role of weight and body dissatisfaction (Young-Hyman et al., 2016; Peterson et al., 2018); other evidence identified different risk profiles of binge eating and purging for adolescent males and females with and without T1D (Neumark-Sztainer et al., 1996) and different predictors for bulimia/food preoccupation in adolescents with T1D (Rohde et al., 2015).

However, it is possible to suppose that the differences between male and female bulimic symptom predictors described by the present findings could also be attributable to the shortcomings in the DEB measures employed. It should be noted that specific items on both DEPS-r and EDI-3RF might be inaccurate in identifying bulimic symptoms in males. For example, items related to purging behaviors focusing on self-induced vomiting and on misuse of laxatives and diuretics (if any) can only detect a small percentage of males who may be more likely to engage in compensatory behaviors such as excessive exercise to lose weight or enhance muscularity (d’Emden et al., 2013). Nonetheless, the exact reasons for lack of significant predictors of bulimic symptoms in male adolescents in the current sample are unknown, and it is unclear whether gender differences exist in the adolescents’ predictors of bulimia, highlighting the need to address this gap.

This study is not without limitations. The major limitation is that collection of the data was from one research patient relied on voluntary participation, and thus generalizability to a general population should be done with caution. The second limitation concerns the use of self-reported measures that may yield imprecise, and possibly inaccurate, ratings of subjective perceptions of behaviors, thoughts, and feelings that might not even be sincerely or fully revealed. It is important for future studies to investigate by using additional body image measures, such as clinical interview, that take into account gender difference and different aspects of the construct. A combination of currently available measures (i.e., silhouette charts to assess body image perception/dissatisfaction and self-report scales to assess body dissatisfaction) to evaluate body image could further enrich the knowledge about the association between body image and T1D and their relationship with DEB.

Finally, given the cross-sectional nature of the results, longitudinal research is needed to further explore the relationship between DEB and clinical and psychological variables.

Despite these limitations, this study has a number of strengths. Given that body image is considered by most authors to be a single, mostly undifferentiated, construct, the major contribution of this study was to measure body image, clearly discriminating between more than one dimension, by taking into account internalization and adhesion of the ideal aspect proposed by the current cultural influences and related perceived pressures. The results of this study offer important contributions to our understanding of the body-related perceptions of youths with T1D, extending previous research by exploring the possible effects of chronic illness on body image.

T1D and insulin therapy may increase risk of weight gain and promote, along with adults’ (parents, doctors, etc.) attention to body size/functioning and frequent emphasis on physical activity, a special focus on the body and thus contribute to the development of body-image problems. Therefore, those patients need to be supported in order not to feel excessively burdened by their illness and its management. This is particularly true in light of current advanced technologies integrated into the daily diabetes care regimen with related rules and calculations (i.e., carbohydrate counting as a meal planning approach with portion control, rules to estimate insulin bolus dosages based on an insulin-to-carbohydrate ratio, setting of the individual’s correction factor, etc.) that may further increase continuous attention to food intake and to body functioning.

Prevention interventions should focus on putative risk factors of DEB onset, taking into account, especially for bulimic symptoms, possible differences between females and males.

Continuity of medical, nutritional, and psychological care through regular clinic attendance is needed in order to maintain positive relationships with clinic staff and to enable continuous monitoring of physical and psychological conditions, especially in patients for whom body image and DEBs are a concern (d’Emden et al., 2013; Young-Hyman et al., 2016; Delamater et al., 2018; American Diabetes Association [ADA], 2020a).

Finally, it should be noted that a high percentage of people with diabetes, especially those with higher BMI and poorer glycemic control, experience stigma (Liu et al., 2017). Since diabetes stigma negatively impacts the management of the disease—and since experience with weight stigma and weight bias internalization is associated with unhealthy eating behaviors, eating pathology, and body image concerns (Durso et al., 2016; Vartanian and Porter, 2016; Brazeau et al., 2018)—proper medical and psychological care should be seen as essential to ensuring the overall health and wellbeing of individuals with T1D.

## Data Availability Statement

The raw data supporting the conclusions of this article will be made available by the authors, without undue reservation, to any qualified researcher.

## Ethics Statement

The studies involving human participants were reviewed and approved by Ethics Committee of University of Campania “Luigi Vanvitelli.” Written informed consent to participate in this study was provided by the participants’ legal guardian/next of kin.

## Author Contributions

The authors contributed to the study as follows: AT designed the study, analyzed the data, and wrote the manuscript. DI supervised this work, designed the study, and contributed to the manuscript. CC, AC, AZ, AP, AB, FC, and EG collected data and contributed to the data analyses and to the manuscript. All authors contributed to the article and approved the submitted version.

## Conflict of Interest

The authors declare that the research was conducted in the absence of any commercial or financial relationships that could be construed as a potential conflict of interest.
